# Design and Synthesis of Novel Nordihydroguaiaretic Acid (NDGA) Analogues as Potential FGFR1 Kinase Inhibitors With Anti-Gastric Activity and Chemosensitizing Effect

**DOI:** 10.3389/fphar.2020.518068

**Published:** 2020-09-11

**Authors:** Qian Chen, Min Zhu, Jingwen Xie, Zhaojun Dong, Fatehi Khushafah, Di Yun, Weitao Fu, Ledan Wang, Tao Wei, Zhiguo Liu, Peihong Qiu, Jianzhang Wu, Wulan Li

**Affiliations:** ^1^ The First Affiliated Hospital of Wenzhou Medical University, Wenzhou, China; ^2^ Chemical Biology Research Center, College of Pharmaceutical Sciences, Wenzhou Medical University, Wenzhou, China; ^3^ Department of Gynecology and Obstetrics, The Second Affiliated Hospital of Wenzhou Medical University, Wenzhou, China

**Keywords:** fibroblast growth factor receptor-1 inhibitor, synthesis, gastric cancer, chemosensitizing effect, nordihydroguaiaretic acid analogues

## Abstract

Aberrant fibroblast growth factor receptor-1 (FGFR1), a key driver promoting gastric cancer (GC) progression and chemo-resistance, has been increasingly recognized as a potential therapeutic target in GC. Hereon, we designed and synthesized a series of asymmetric analogues using **Af23** and NDGA as lead compounds by retaining the basic structural framework (bisaryl-1,4-dien-3-one) and the unilateral active functional groups (3,4-dihydroxyl). Thereinto, **Y14** showed considerable inhibitory activity against FGFR1. Next, pharmacological experiments showed that **Y14** could significantly inhibit the phosphorylation of FGFR1 and its downstream kinase AKT and ERK, thus inhibiting the growth, survival, and migration of gastric cancer cells. Furthermore, compared with 5-FU treatment alone, the combination of **Y14** and 5-FU significantly reduced the phosphorylation level of FGFR1, and enhanced the anti-cancer effect by inhibiting the viability and colony formation in two gastric cancer cell lines. These results confirmed that **Y14** exerted anti-gastric activity and chemosensitizing effect by inhibiting FGFR1 phosphorylation and its downstream signaling pathway *in vitro*. This work also provides evidence that **Y14**, an effective FGFR1 inhibitor, could be used alone or in combination with chemotherapy to treat gastric cancer in the future.

## Introduction

Fibroblast growth factor receptor-1 (FGFR1), known as one member of receptor tyrosine kinases (RTKs), is considered as an attractive target for cancer treatment increasingly (Ng et al., 2018; Chen et al., 2019). FGFR1 consists of three conventional key domains structurally, the extracellular domain with three immunoglobulin subunits, a single-pass transmembrane domain and two intracellular kinase domains (Mohammadi et al., 2005). After binding to ligands, FGFR1 undergoes dimerization and trans-autophosphorylation, which activates an array of downstream signaling pathways, including the mitogen activated protein kinase (MAPK), phosphoinositide 3-kinase (PI3K)/AKT, phospholipase Cγ (PLCγ), and signal transducer and activator of transcription 3 (STAT3) (Carter et al., 2015). It has been widely proved that FGFR1 dysregulation is closely correlated with the development of various cancers by promoting cell proliferation, migration, differentiation, and survival (Babina and Turner, 2017; Wang et al., 2018). FGFR1 is also implicated in a higher risk of distant metastasis and chemo-resistance of various cancer (Cao et al., 2017; Jang et al., 2017), such as malignant lymphoma (Cao et al., 2014), breast cancer (Turner et al., 2010; Golfmann et al., 2018), and gastric cancer (Chen et al., 2014; Schafer et al., 2015; Shimizu et al., 2018). In recent years, growing evidence indicates that FGFR1 might be a promising therapeutic target for the treatment of gastric cancer (Gu et al., 2019; Xie et al., 2019). It has been reported that high expression of FGFR1 was shown by 37% (40/109) in diffuse-type gastric cancer sample and 38% (38/100) in intestinal-type gastric cancer sample (Inokuchi et al., 2017). Shin et al. found that FGFR1 mRNA and protein expression were upregulated in human gastric cancer versus their normal counterparts (Shin et al., 2000). Compared with FGFR1 non-amplified gastric adenocarcinoma, FGFR1-amplified cases are associated with poor 10-year survival (*p* = 0.047) and a higher rate of distant metastasis (*p* = 0.025) (Schafer et al., 2015). Furthermore, the result of qRT-PCR showed that FGFR1 was significantly up-regulated in drug-resistant gastric cancer cell lines SGC-7901/VCR and SGC-7901/ADR, which is in accordance with the mRNA profiling results (Chen et al., 2014).

In recent years, development of FGFR1 inhibitors targeting gastric cancer have attracted extensive attentions (Gu et al., 2019; Xie et al., 2019). AZD4547, a pan FGFR1/2/3 inhibitor, has been completed a phase II clinical trial in advanced GC patients (NCT01795768) (Xie et al., 2013). Another strong FGFR1/2/3 inhibitor, BGJ398, is under a phase I study in patients with untreated advanced gastrointestinal stromal tumor (NCT02257541) (Schmidt et al., 2015). Although much progress has been made in this field, no FDA-approved drug is available to treat gastric cancer by inhibiting FGFR1. Currently, the majority of FGFR1 inhibitors belong to ATP competitive inhibitors, which are designed to bind and inhibit the ATP-binding pocket of FGFR1. Owing to the high similarity of ATP-binding pocket among RTKs members, the design strategy of FGFR1 inhibitor based on receptor structure seems to be faced with a big conundrum that such inhibitors often lead to severe side effects and off-target toxicities by inhibiting other RTKs (Ho et al., 2014). To discover novel ATP-independent kinase inhibitors by taking natural products as lead compound develops a considerable alternative gradually (Liu et al., 2013). A few ATP-independent FGFR1 inhibitors have been synthesized, which exhibited anti-cancer activity *in vitro* or *in vivo* (Wang et al., 2014; Wu et al., 2017a; Ying et al., 2017). In our previous study, a series of symmetric structural analogues of nordihydroguaiaretic acid (NDGA), a phenolic compound isolated from the *creosote bush*, was constructed and it was found that **Af23** (IC_50_ = 1.4 μM) was a non-ATP-competitive FGFR1 inhibitor with excellent anti-lung cancer activity *in vitro* and *in vivo* (Wu et al., 2015).

Gastric cancer has always been one of the main causes of cancer-related death in the world, especially in Asia. Although the prognosis is poor, chemotherapy remains the main treatment for patients with advanced gastric cancer. Targeted therapy is an important approach to improve the therapeutic effect of advanced gastric cancer, but only a few drugs targeting HER2 or VEGFR2 have been approved for clinical application (Casak et al., 2015; Ock et al., 2015). Moreover, some phase 1/II clinical trials have proved the safety and efficacy of targeted FGFR1 in the treatment of advanced gastric cancer (NCT01795768, NCT02257541). Therefore, in this study, a series of asymmetric analogues were designed and synthesized using **Af23** and NDGA as lead compounds. Among them, compound **Y14** was successfully screened out as a novel NDGA-based FGFR1 inhibitor with favorable anti-gastric cancer activity.

## Materials and Methods

### Chemistry

All reagents and solvents for the synthesis were purchased from Sigma-Aldrich (USA) and Aladdin (China). The reaction was monitored by column chromatography (200–300 mesh) on silica gel GF254. Melting points were measured and were uncorrected on a Fisher-Johns melting apparatus. Mass spectrometry (MS) was performed by an Agilent 1100 LC-MS (Agilent, Palo Alto, CA, USA).

The ^1^H-NMR and ^13^C-NMR spectra data were recorded on a 600 or 500 MHz spectrometer from Bruker Corporation (Switzerland) with TMS as an internal standard.

### Synthesis of the Intermediates 3

As our previous report (Wu et al., 2017b), a solution of tetrahydro-4H-pyran-4-one (**1**, 20 mmol), morpholine (**2**, 30 mmol) and 4-methylbenzenesulfonic acid (200 mg) in cyclohexane (20 ml) was heated to reflux at 90°C for 4 h. After cooling to room temperature, the mixture was washed with water, dried, and concentrated to obtain the enamine intermediates **3** as a brown oil, which was directly used for the next reaction.

### Synthesis of the Intermediates 5

A mixture of **3** (10 mmol) and 3,4-dihydroxybenzaldehyde (**4**, 10 mmol) was dissolved in ethanol (20 ml) and the resulting solution was stirred at 78°C for 2 h. The residue was concentrated under vacuum and purified by column chromatography to give an orange powder. The powder was re-dissolved in ethanol (10 ml), and 10% HCl solution (4 ml) was added. After being stirred at room temperature for 3 h, distilled water (20 ml) was poured into the reaction flask. A light-yellow precipitate of **5** was collected and washed with water. 1H-NMR (DMSO-d6), δ: 9.719 (s, 1H, 3-OH), 9.334 (s, 1H, 4-OH), 7.435 (s, 1H, Ar-CH=C), 6.922 (d, J=7.8 HZ, 2H, H-2, H-6), 6.860 (d, J=9.6 HZ, 1H, H-5), 4.092 (t, J=6.0 HZ, 2H, O-CH2-C), 3.471 (s, 1H, O-CH2-CH2), 2.647 (t, J=6.0 HZ, 3H, O-CH2-CH2).

### General Procedure for Synthesis of Compounds Y1–Y18

Various benzaldehydes (2 mmol) were dissolved into a suspension of the intermediates **5** (2 mmol) in ethanol (10 ml). Subsequently, HCl gas was bubbled into the mixture for 30 min, and the resulting mixture was stirred at 50–70 °C for 2–3 h. After removal of ethanol, water (10 ml) and ethyl ester (20 ml) were poured into the reaction solution. The organic solvent was removed under reduced pressure and the residue was purified by chromatography to obtain the products **Y1–Y18**.

(3*E*,5*E*)-3-(3,4-dihydroxybenzylidene)-5-(4-(dimethylamino)benzylidene)dihydro-2H-pyran-4(3H)-one **(Y1)**

Yellowish-brown powder, Yield: 40.7%, mp. 210.1–213.3 °C. ^1^H-NMR (DMSO-d_6_) δ: 7.564 (s, 1H, Ar-CH=C), 7.470 (s, 1H, Ar’-CH=C), 7.314 (d, *J=*8.4 Hz, 2H, H-2’, H-6’), 6.860 (d, *J=*7.2 Hz, H-6), 6.824-6.838 (m, 3H, H-2, H-3’, H-5’), 6.762 (dd, *J=*1.8 Hz, 8.4 Hz, 1H, H-5), 4.585 (d, *J=*12.0 Hz, 4H, CH_2_-O-CH_2_), 3.006 (s, 6H, CH_3_-N-CH_3_). ^13^C-NMR (CDCl_3_-d_6_), δ: 189.68, 163.66, 151.53, 149.36, 144.84, 143.23, 142.31, 134.04, 131.30, 129.87, 128.60, 123.10, 119.85, 119.30, 111.29, 110.39, 58.45, 56.01. ESI-MS m/z: 349.5 (M-1)^-^, calcd for C_21_H_21_NO_4_: 351.1.

(3*E*,5*E*)-3-(3,4-dihydroxybenzylidene)-5-(4-hydroxy-3-methoxybenzylidene)dihydro-2H-pyran-4(3H)-one **(Y2)**

Gray-yellow powder, Yield: 18.9%, mp. 228.1–231.1 °C. ^1^H-NMR (DMSO-d_6_) δ: 9.67 (s, 1H, 4-OH), 9.602 (s, 1H, 3-OH), 9.229 (s, 1H, 4’-OH), 7.581 (s, 1H, Ar-CH=C), 7.491 (s, 1H, Ar’-CH=C), 7.011 (d, *J=*7.2 Hz, 1H, H-6), 6.871 (d, *J=*3.0 Hz, 2H, H-5, H-5’), 6.828 (dd, *J=*1.8 Hz, 7.8 HZ, 2H, H-2, H-2’), 6.782 (dd, *J=*1.8 Hz, 8.4Hz, 1H, H-6’), 4.897 (d, *J=*12.0 Hz, 4H, CH_2_-O-CH_2_), 3.823 (s, 3H, 3’-OCH_3_). ^13^C-NMR (DMSO-d_6_), δ: 184.36, 148.49, 147.60, 147.55, 145.34, 135.29, 135.17, 130.87, 130.47, 125.83, 124.59, 123.70, 117.77, 115.92, 115.73, 114.87, 67.83, 67.74, 55.66. ESI-MS m/z: 352.9 (M-1)^-^, calcd for C_20_H_18_O_6_: 354.1.

(3*E*,5*E*)-3-(3,4-dihydroxybenzylidene)-5-(4-methoxybenzylidene)dihydro-2H-pyran-4(3H)-one **(Y3)**

Kelly powder, Yield: 68.0%, mp. 229.4–232.2 °C. ^1^H-NMR (DMSO-d_6_) δ: 9.618 (s, 1H, 4-OH), 9.235 (s, 1H, 3-OH), 7.603 (s, 1H, Ar-CH=C), 7.500 (s, 1H, Ar’-CH=C), 7.398 (d, *J=*8.4 Hz, 2H, H-2’, H-6’), 7.033 (d, *J=*8.4 Hz, 2H, H-3’, H-5’), 6.833 (d, *J=*2.4 Hz, 1H, H-2), 6.822 (s, 1H, H-5), 6.786 (dd, *J=*1.8 Hz, 8.4 Hz, 1H, H-6), 4.871(d, *J=*12.0 Hz, CH_2_-O-CH_2_), 3.814 (s, 3H, 4’-OCH_3_). ^13^C-NMR (DMSO-d_6_), δ: 184.40, 160.24, 147.62, 145.36, 135.51, 134.26, 132.49, 131.61, 130.38, 126.87, 125.78, 123.75, 117.81, 115.92, 114.34, 67.88, 67.70, 55.30. ESI-MS m/z: 336.9 (M-1)^-^, calcd for C_20_H_18_O_5_: 338.1.

(3*E*,5*E*)-3-(3,4-dihydroxybenzylidene)-5-(2-fluorobenzylidene)dihydro-2H-pyran-4(3H)-one **(Y4)**

Kelly powder, Yield: 11.1%, mp. 211.1–213.5 °C. ^1^H-NMR (DMSO-d_6_) δ: 9.681 (s, 1H, 4-OH), 9.266 (s, 1H, 3-OH), 7.683 (s, 1H, Ar’-CH=C), 7.546 (s, 1H, Ar-CH=C), 7.501 (q, *J=*6.6 Hz, 1H, H-4’), 7.288-7.371 (m, 3H, H-3’, H-5’, H-6’), 6.855 (d, *J=*1.2 Hz, 1H, H-2), 6.840 (d, *J=*8.4 Hz, 1H, H-5), 6.815 (dd, *J=*1.8 Hz, 8.4 Hz, 1H, H-6), 4.890 (s, 2H, Ar’-C=C-CH_2_), 4.788 (s, 2H, Ar-C=C-CH_2_). ^13^C-NMR (DMSO-d_6_), δ: 184.47, 147.77, 145.38, 136.03, 133.57, 133.08, 132.85, 132.78, 130.88, 130.86, 130.16, 125.68, 123.91, 117.87, 115.95, 115.85, 115.68, 67.96, 67.46. ESI-MS m/z: 324.8 (M-1)^-^, calcd for C_19_H_15_FO_4_: 326.1.

(3*E*,5*E*)-3-(3,4-dihydroxybenzylidene)-5-(4-hydroxybenzylidene)dihydro-2H-pyran-4(3H)-one **(Y5)**

Gray-yellow powder, Yield: 65.1%, mp. 285.4–288.8 °C. ^1^H-NMR (DMSO-d_6_) δ: 10.057 (s, 1H, 4-OH), 9.599 (s, 1H, 4’-OH), 9.225 (s, 1H, 3-OH), 7.562 (s, 1H, Ar-CH=C), 7.485 (s, 1H, Ar’-CH=C), 7.289 (d, *J=*8.4 Hz, 2H, H-2’, H-6’), 6.856 (d, *J=*8.4 Hz, 2H, H-3’, H-5’), 6.818–6.832 (m, 2H, H-2, H-5), 6.777 (dd, *J=*1.8 Hz, 7.8 Hz, H-6), 4.857 (d, *J=*7.2 Hz, 4H, CH_2_-O-CH_2_). ^13^C-NMR (CDCl_3_-d_6_), δ: 189.23, 163.65, 152.11, 145.68, 141.49, 134.93, 130.98, 130.40, 129.68, 128.40, 122.77, 119.26, 116.66, 111.88, 40.08. ESI-MS m/z: 322.9 (M-1)^-^, calcd for C_19_H_16_O_5_: 324.1.

(3*E*,5*E*)-3-(3,4-dihydroxybenzylidene)-5-(4-fluorobenzylidene)dihydro-2H-py-ran-4(3H)-one **(Y6)**

Yellow powder, Yield: 13.8%, mp. 223.4–227.3 °C. ^1^H-NMR (DMSO-d_6_) δ: 9.651 (s, 1H, 4-OH), 9.249 (s, 1H, 3-OH), 7.631 (s, 1H, Ar’-CH=C), 7.519 (s, 1H, Ar-CH=C), 7.485–7.509 (m, 2H, H-5’, H-6’), 7.291–7.320 (m, 2H, H-2’, H-4’), 6.842 (d, *J=*1.8 Hz, 1H, H-2), 6.834 (d, *J=*8.4 Hz, 1H, H-5), 6.801 (dd, *J=*1.8 Hz, 8.4 Hz, 1H, H-6). 4.873 (d, *J=*1.2 Hz, 4H, CH_2_-O-CH_2_). ^13^C-NMR (DMSO-d_6_), δ: 184.47, 147.77, 145.38, 136.03, 133.57, 133.08, 132.85, 132.78, 130.88, 130.86, 130.16, 125.68, 123.91, 117.87, 115.95, 115.85, 115.68, 67.96, 67.46. ESI-MS m/z: 324.8 (M-1)^-^, calcd for C_19_H_15_FO_4_: 326.1.

(3*E*,5*E*)-3-(3,4-dihydroxybenzylidene)-5-(3-hydroxybenzylidene)dihydro-2H-pyran-4(3H)-one **(Y7)**

Yellow powder, Yield: 23.6%, mp. 332.8–336.3 °C. ^1^H-NMR (DMSO-d_6_) δ: 9.645 (s, 2H, 3-OH, 3’-OH), 9.247 (s, 1H, 4-OH), 7.541 (s, 1H, Ar-CH=C), 7.510 (s, 1H, Ar’-CH=C), 7.266 (t, *J=*7.8 Hz, 1H, H-5’), 6.826–6.850 (m, 4H, H-5, H-6, H-4’, H-6’), 6.803 (d, *J=*1.8 Hz, 1H, H-2), 6.786 (d, *J=*1.8 Hz, 1H, H-2’), 4.867 (d, *J=*6.6 Hz, 4H, CH_2_-O-CH_2_). ^13^C-NMR (CDCl_3_-d_6_), δ: 189.23, 163.65, 152.11, 145.68, 141.49, 134.93, 130.98, 130.40, 129.68, 128.40, 122.77, 119.26, 116.66, 111.88, 40.08. ESI-MS m/z: 322.8 (M-1)^-^, calcd for C_19_H_16_O_5_: 324.1.

(3*E*,5*E*)-3-(3,4-dihydroxybenzylidene)-5-(2,4,6-trimethoxybenzylidene)dihydro-2H-pyran-4(3H)-one **(Y8)**

Yellow powder, Yield: 54.9%, mp. 215.3–217.5 °C. ^1^H-NMR (DMSO-d_6_) δ: 9.608 (s, 1H, 4-OH), 9.233 (s, 1H, 3-OH), 7.511 (s, 1H, Ar-CH=C), 7.477 (s, 1H, Ar-CH=C), 6.820–6.833 (m, 2H, H-2, H-5), 6.782 (dd, *J=*1.8 Hz, 8.4 Hz, H-6), 6.293 (s, 2H, H-3’, H-5’), 4.842 (s, 2H, Ar-C=C-CH_2_), 4.275 (s, 2H, Ar’-C=C-CH_2_), 3.832 (s, 3H, 4’-OCH_3_), 3.806 (s, 6H, 2’-OCH_3_, 6’-OCH_3_). ^13^C-NMR (DMSO-d_6_), δ: 184.64, 162.42, 159.00, 147.27, 145.34, 136.47, 135.63, 127.07, 125.65, 122.73, 117.90, 115.66, 104.25, 99.40, 94.25, 91.71, 90.83, 68.30, 60.79, 60.33, 56.15, 55.48. ESI-MS m/z: 396.9 (M-1)^-^, calcd for C_22_H_22_O_7_: 398.1.

(3*E*,5*E*)-3-(3,4-dihydroxybenzylidene)-5-(3-hydroxy-4-methoxybenzylidene)dihydro-2H-pyran-4(3H)-one **(Y9)**

Dark brown powder, Yield: 31.6%, mp. 218.7–221.1 °C. ^1^H-NMR (DMSO-d_6_) δ: 7.504 (s, 1H, Ar-CH=C), 7.488 (s, 1H, Ar’-CH=C), 7.011 (d, *J=*8.4 Hz, 1H, H-5), 6.889 (dd, *J*=1.8 Hz, 8.4 Hz, 1H, H-6), 6.853 (d, *J=*1.8 Hz, 1H, H-2), 6.821–6.834 (m, 2H, H-2’, H-5’), 6.779 (dd, *J=*1.8 Hz, 8.4 Hz, 1H, H-6’), 4.862 (s, 4H, CH_2_-O-CH_2_), 3.820 (s, 3H, 4’-OCH_3_). ^13^C-NMR (DMSO-d_6_), δ: 184.36, 148.49, 147.60, 147.55, 145.34, 135.29, 135.17, 130.87, 130.47, 125.83, 124.59, 123.70, 117.77, 115.92, 115.73, 114.87, 67.83, 67.74, 55.66. ESI-MS m/z: 352.9 (M-1)^-^, calcd for C_20_H_18_O_6_: 354.1.

(3*E*,5*E*)-3-(3,4-dihydroxybenzylidene)-5-(2,4-dimethoxybenzylidene)dihydro-2H-pyran-4(3H)-one **(Y10)**

Yellow powder, Yield: 93.4%, mp. 233.0–236.4 °C. ^1^H-NMR (DMSO-d_6_) δ: 9.609 (s, 1H, 4-OH), 9.229 (s, 1H, 3-OH), 7.829 (s, 1H, Ar’-CH=C), 7.483 (s, 1H, Ar-CH=C), 7.077 (d, *J=*8.4 Hz, 1H, H-6’), 6.826 (d, *J=*7.8 Hz, 2H, H-2, H-5), 6.783 (dd, *J*=1.8 Hz, 7.8 Hz, 1H, H-6), 6.652 (d, *J=*1.8 Hz, 1H, H-3’), 6.604 (dd, *J*=1.8 Hz, 8.4 Hz, 1H, H-5’). 4.762–4.852 (m, 4H, CH_2_-O-CH_2_), 3.860 (s, 3H, 2’-OCH_3_), 3.825 (s, 3H, 4’-OCH_3_). ^13^C-NMR (DMSO-d_6_), δ: 184.41, 162.18, 159.67, 147.58, 145.35, 135.48, 131.56, 131.17, 130.45, 129.62, 125.84, 123.74, 117.81, 115.92, 115.64, 105.36, 98.44, 68.07, 67.74, 55.72, 55.44. ESI-MS m/z: 366.9 (M-1)^-^, calcd for C_21_H_20_O_6_: 368.1.

(3*E*,5*E*)-3-(3,4-dihydroxybenzylidene)-5-(2-methoxybenzylidene)dihydro-2H-pyran-4(3H)-one **(Y11)**

Green powder, Yield: 88.4%, mp. 184.7–187.4 °C. ^1^H-NMR (DMSO-d_6_) δ: 9.640 (s, 1H, 4-OH), 9.245 (s, 1H, 3-OH), 7.842 (s, 1H, Ar’-CH=C), 7.509 (s, 1H, Ar-CH=C), 7.426 (td, *J*=1.8 Hz, 7.8 Hz, 1H, H-4’), 7.137 (dd, *J=*1.2 Hz, 8.4 Hz, 1H, H-6’), 7.105 (d, *J=*8.4 Hz, 1H, H-5), 7.014 (t, *J=*7.8 Hz, 1H, H-5’), 6.827–6.843 (m, 2H, H-2, H-3’), 6.801 (dd, *J*=1.8 Hz, 8.4 Hz, 1H, H-6), 4.868 (s, 2H, Ar-C=C-CH_2_), 4.757(s, 2H, Ar’-C=C-CH_2_). ^13^C-NMR (DMSO-d_6_), δ; 184.40, 160.24, 147.62, 145.36, 135.51, 134.26, 132.49, 131.61, 130.38, 126.87, 125.78, 123.75, 117.81, 115.92, 114.34, 67.88, 67.70, 55.30. ESI-MS m/z: 336.5 (M-1)^-^, calcd for C_20_H_18_O_5_: 338.1.

(3*E*,5*E*)-3-(3,4-dihydroxybenzylidene)-5-(4-(piperidin-1-yl)benzylidene)dihydro-2H-pyran-4(3H)-one **(Y12)**

Gray powder, Yield: 38.1%, mp. >300 °C. ^1^H-NMR (DMSO-d_6_) δ: 7.587(s, 1H, Ar-CH=C), 7.498 (s, 1H, Ar’-CH=C), 7.424 (s, 3H, H-2, H-2’, H-6’), 6.838 (d, *J=*8.4 Hz, 2H, H-3’, H-5’), 6.783 (dd, *J=*1.8 Hz, 8.4 Hz, 1H, H-6), 4.870 (d, *J=*13.8 Hz, 4H, CH_2_-O-CH_2_), 3.415(s, 4H, CH_2_-N-CH_2_). ^13^C NMR (CDCl_3_-d_6_), δ: 189.05, 163.63, 153.54, 144.83, 141.92, 134.15, 130.84, 130.42, 129.93, 128.65, 121.24, 119.33, 105.89, 60.97, 56.30. ESI-MS m/z: 390.0 (M-1)^-^, calcd for C_24_H_25_NO_4_: 391.1.

(3*E*,5*E*)-3-(3,4-dihydroxybenzylidene)-5-(4-morpholinobenzylidene)dihydro-2H-pyran-4(3H)-one **(Y13)**

Orange yellow powder, Yield: 54.6%, mp. >300 °C. ^1^H-NMR (DMSO-d_6_) δ: 9.592 (s, 1H, 4-OH), 9.224 (s, 1H, 3-OH), 7.563 (s, 1H, Ar-CH=C), 7.480 (s, 1H, Ar’-CH=C), 7.318 (d, *J=*9.0 Hz, 2H, H-2’,H-6’), 7.006 (d, *J=*8.4 Hz, 2H, H-3’, H5’), 6.802–6.831 (m, 2H, H-2, H-5), 6.772 (dd, *J*=1.2 Hz, 7.8 Hz, 1H, H-6), 4.867 (d, *J=*19.8 Hz, 4H, CH_2_-O-CH_2_), 3.736 (d, *J=*4.8 Hz, 4H, CH_2_-O-CH_2_), 3.241 (t, *J=*4.8 Hz, 4H, CH_2_-N-CH_2_). ^13^C NMR (CDCl_3_-d_6_), δ: 185.27, 151.54, 135.89, 132.54, 132.37, 130.61, 125.96, 114.50, 68.76, 66.66, 48.02. ESI-MS m/z: 391.7 (M-1)^-^, calcd for C_23_H_23_NO_5_: 393.2.

(3*E*,5*E*)-3-(3,4-dihydroxybenzylidene)-5-(2,3-dimethoxybenzylidene)dihydro-2H-pyran-4(3H)-one **(Y14)**

Light green powder, Yield: 25.3%, mp. 138.5–141.1 °C. ^1^H-NMR (DMSO-d_6_) δ: 9.653 (s, 1H, 4-OH), 9.253 (s, 1H, 3-OH), 7.796 (s, 1H, Ar-CH=C), 7.521 (s, 1H, Ar’-CH=C), 7.122–7.158 (m, 2H, H-2, H-5), 6.836 (d, *J=*9.6 Hz, 2H, H-6, H-6’), 6.808 (td, *J*=1.8 Hz, 7.8 Hz, 1H, H-5’), 6.776 (dd, *J=*1.8 Hz, 7.2Hz, 1H, H-4’), 4.870 (s, 2H, Ar-C=C-CH_2_), 4.762 (s, 2H, Ar’-C=C-CH_2_), 3.836 (s, 3H, 2’-OCH_3_), 3.736 (s, 3H, 3’-OCH_3_). ^13^C NMR (DMSO-d_6_), δ: 187.33, 168.62, 163.61, 143.54, 143.43, 141.00, 131.53, 129.94, 127.76, 123.33, 118.77, 118.47, 117.97, 117.83, 116.97, 116.83, 56.00, 54.88, 54.83. ESI-MS m/z: 366.8 (M-1)^-^, calcd for C_21_H_20_O_6_: 368.1.

(3*E*,5*E*)-3-(3,4-dihydroxybenzylidene)-5-(2,5-dimethoxybenzylidene)dihydro-2H-pyran-4(3H)-one **(Y15)**

Kelly powder, Yield: 63.5%, mp. 179.1–182.7 °C. ^1^H-NMR (DMSO-d_6_) δ: 9.646 (s, 1H, 4-OH), 9.251 (s, 1H, 3-OH), 7.788 (s, 1H, Ar-CH=C), 7.508 (s, 1H, Ar’-CH=C), 7.034 (d, *J=*9.0 Hz,1H, Ar-H5), 6.999 (dd, *J*=2.4 Hz, 9.0 Hz, Ar-H6), 6.827–6.841 (m, 2H, Ar-H2, Ar-H3’), 6.797 (dd, *J*=1.8 Hz, 8.4 Hz, Ar-H4’), 6.679 (d, *J=*3.0 Hz, 1H, Ar-H6’), 4.823 (d, *J*=12.0 Hz, 4H, CH_2_-O-CH_2_), 3.800 (s, 3H, 2’-OCH_3_), 3.743 (s, 3H, 5’-OCH_3_). ^13^C-NMR (DMSO-d_6_), δ: 187.33, 168.62, 163.61, 143.54, 143.43, 141.00, 131.53, 129.94, 127.76, 123.33, 118.77, 118.47, 117.97, 117.83, 116.97, 116.83, 56.00, 54.88, 54.83. ESI-MS m/z: 366.5 (M-1)^-^, calcd for C_21_H_20_O_6_: 368.1.

(3*E*,5*E*)-3-(4-(diethylamino)benzylidene)-5-(3,4-dihydroxybenzylidene)dihydro-2H-pyran-4(3H)-one **(Y16)**

Yellowish-brown powder, Yield: 21.1%, mp. 199.9–202.2 °C. ^1^H-NMR (DMSO-d_6_) δ: 7.566 (s, 1H, Ar-CH=C), 7.478 (s, 1H, Ar’-CH=C), 7.319 (s, 2H, H-2’, H-6’), 6.809–6.837 (m, 3H, H-3’, H-5’, H-2), 6.769 (d, *J=*7.8 Hz, 1H, H-5), 6.746 (dd, *J=*2.4 Hz, 8.4 Hz, 1H, H-6), 4.854 (d, *J=*8.4 Hz, 4H, CH_2_-O-CH_2_), 3.443 (s, 4H, CH_2_-N-CH_2_), 1.096 (t, *J=*7.2 Hz, 6H, CH_3_×2). ^13^C-NMR (CDCl_3_-d_6_), δ: 189.68, 166.34, 151.53, 149.49, 148.73, 144.84, 143.23, 142.31, 134.04, 131.30, 129.87, 129.16, 128.60, 127.83, 123.10, 119.85, 119.30, 118.44, 111.29, 110.88, 110.39, 58.45, 56.01, 18.40. ESI-MS m/z: 377.8 (M-1)^-^, calcd for C_23_H_25_NO_4_: 379.2.

(3*E*,5*E*)-3-(3,4-dihydroxybenzylidene)-5-(3,4,5-trimethoxybenzylidene)dihydro-2H-pyran-4(3H)-one **(Y17)**

Kelly powder, Yield: 48.6%, mp. 180.7–183.9 °C. ^1^H-NMR (DMSO-d_6_) δ: 9.642 (s, 1H, 3-OH), 9.252 (s, 1H, 4-OH), 7.603 (s, 1H, Ar-CH=C), 7.516 (s, 1H, Ar’-CH=C), 6.841 (d, *J=*7.2 Hz, 1H, Ar-H2), 6.836 (d, *J=*7.2 Hz, 1H, Ar-H5), 6.780 (dd, *J*=1.8 Hz, 8.4 Hz, 1H, Ar-H6), 6.721 (s, 2H, Ar-H2’, Ar-H6’), 4.913 (d, *J=*12.0 Hz, 4H, CH_2_-O-CH_2_), 3.976 (s, 6H, 3’-OCH_3_, 5’-OCH_3_), 3.327 (s, 3H, 4’-OCH_3_). ^13^C NMR (DMSO-d_6_). δ: 184.64, 162.42, 159.00, 147.27, 145.34, 136.47, 135.63, 127.07, 125.65, 122.73, 117.90, 115.66, 104.25, 99.40, 94.25, 91.71, 90.83, 68.30, 60.79, 60.33, 56.15, 55.48. ESI-MS m/z: 396.6 (M-1)^-^, calcd for C_22_H_22_O_7_: 398.1.

(3*E*,5*E*)-3-(3,4-dihydroxybenzylidene)-5-(3,4-dimethoxybenzylidene)dihydro-2H-pyran-4(3H)-one **(Y18)**

Brown powder, Yield: 59.4%, mp. 226.4–229.3 °C. ^1^H-NMR (DMSO-d_6_) δ: 9.618 (s, 1H, 3-OH), 9.237 (s, 1H, 4-OH), 7.610 (s, 1H, Ar-CH=C), 7.504 (s, 1H, Ar’-CH=C), 7.029–7.057 (m, 2H, H-2, H-5), 6.989 (dd, *J*=1.8 Hz, 8.4 Hz, 1H, H-6), 6.824-6.838 (m, 2H, H-2’, H-5’), 6.788 (dd, *J*=1.8 Hz, 8.4 Hz, 1H, H-6’), 4.891 (d, *J=*12.0 Hz, 4H, CH_2_-O-CH_2_), 3.812 (s, 6H, 3’-OCH_3_, 4’-OCH_3_). ^13^C NMR (DMSO-d_6_), δ: 187.33, 168.62, 163.61, 143.54, 143.43, 141.00, 131.53, 129.94, 127.76, 123.33, 118.77, 118.47, 117.97, 117.83, 116.97, 116.83, 56.00, 54.88, 54.83. ESI-MS m/z: 366.9 (M-1)^-^, calcd for C_21_H_20_O_6_: 368.1.

### Cell-Free FGFR1 Kinase Assays

Using the method described in our previous paper (Ying et al., 2017), the inhibitory activity of **Af23** and compounds **Y1–Y18** on FGFR1 kinase at a concentration of 20 μM was determined by Caliper Mobility Shift Assay. Staphylococcin was used as positive control and 10 mM EDTA as blank control. All conversion data were collected on Caliper EZ reader (Hopkinton, MA).

### Molecular Docking

The binding sites of FGFR1 with **Y14** and **Af23** were studied by using AutoDock (version 4.2.6) software (Morris et al., 2009). The crystallographic co-ordinates for human FGFR1 (PDB ID: 3RHX, 4UWC, 5EW8) were retrieved from the Protein Data Bank (PDB). Firstly, PyMOL software was used to remove water molecules and other ligands to prepare protein structure (Alexander et al., 2011). A grid box with a size of 60 × 60 × 60 dimensions and a spacing of 0.375 Å was set up, covering almost the entire FGFR1 binding site. All docking calculations were used the Lamarckian genetic algorithm (LGA) with 50 LGA runs. Other parameters are set by default. AutoDockTools (version 1.5.6) and PyMol was used to analyze the docking results (Morris et al., 2009).

### Molecular Dynamics (MD) Simulations

The dynamic interaction patterns of two complexes (**Y14**/FGFR1 and **Af23**/FGFR1) were studied by the MD simulations. Using the LEaP module of AMBER 11, ff99SB and GAFF force fields were used as the parameters for inhibitors (**Y14** and **Af23)** and FGFR1, respectively. The atomic charges of the inhibitors were fitted by the restrained electrostatic potential (RESP) fitting procedure based on the electrostatic potentials calculated by Hartree-Fock (HF) method with 6-31G (d) basis set in the Gaussian 09 package. The two systems were neutralized by adding an appropriate number of sodium ions and were solvated in a box of TIP3P water molecules with a hydration shell of 10 Å. Subsequently, an equilibration protocol was carried out including an initial minimization of the water box of 5,000 steps (2,500 steps for the steepest descent and 2,500 steps in the conjugate gradient). The system was heated to 300 K with constant volume by using a time constant for the heat bath with coupling time of 100 ps. The whole system was equilibrated at 100 ps at a constant pressure of 1 atm, and finally was equilibrated for 50 ns without any restrains. Periodic boundary conditions were employed in the whole process. The SHAKE method (Bailey and Lowe, 2009) was used to constrain hydrogen atoms and the time step was set to 2 fs. Long-range electrostatic interactions were handled by the Particle Mesh Ewald (PME) method (Sagui and Darden, 1999) and non-bonded interactions were truncated at 8.0 Å. The coordinates were recorded every 10 ps. All MD simulations were performed using the AMBER 11 software package under the same protocol.

### Binding Free Energy Calculations and Decomposition Analysis

The binding free energy of the complex was evaluated by the molecular mechanics/generalized Born surface area (MM/GBSA) method in AMBER 11 (Hou et al., 2011). For each system, total 500 snapshots were extracted from the last 10 ns MD stable trajectory every 20 ps for the calculations. The binding free energy (ΔG_bind_) was calculated according to the equation (1):

(1) $$\Delta G_{bind}=G_{complex}-(G_{receptor}+G_{ligand})$$

where G_complex_, G_receptor_ and G_ligand_ are the free energy of complex (**Y14**/FGFR1or **Af23**/FGFR1), FGFR1 and inhibitor molecules, respectively. The free energy (G) of complex, FGFR1 or inhibitor was calculated using the following equations (2-4) based on the average of snapshots taken from the MD trajectories.

(2) $$G=H-\text{TS}\approx (E_{gas}+G_{sol}-TS)$$

(3) $$E_{gas}=E_{int}+E_{vdw}+E_{elec}$$

(4) $$G_{sol}=G_{GB}+G_{SA}$$

where E_gas_ is the molecular mechanics free energy in the gas phase, including the internal energy (E*_int_*), the electrostatic (G_elec_), and van der Waals (G_vdw_) interactions. The solvation free energy (G_sol_) were composed of polar (G_GB_) and non-polar (G_SA_) contributions. TS represents the absolute temperature (T) multiplied by the entropy (S).

Finally, the contribution of each residue in FGFR1 to the total binding free energy was evaluated using the MM/GBSA free energy decomposition analysis in AMBER11. The binding interaction between residue-inhibitor pair only taked into account molecular mechanics free energy and solvation free energy.

### Cell Culture

Human gastric cancer SGC-7901 and BGC-823 cells were purchased from China Center for Type Culture Collection (Wuhan, China). All cells were maintained in RMPI-1640 media (Gibco) supplemented with 10% FBS (Gibco) and 1% Penicilin Streptomycin (Gibco) at 37°C in a humidified atmosphere containing 5% CO_2_.

### Western Blot

Human gastric cancer SGC-7901 and BGC-823 cells were seeded in 6-well plates at a density of 3x10^5^ cells/well and adhere overnight. After incubation with DMSO, **Y14** (10, 20 μM), 20 μM **Af23** and NDGA for 2 h, FGF2 (40 ng/ml) was added for 15 min. As for the combination of 5-FU and **Y14**, SGC-7901 and BGC-823 cells were seeded in 6-well plates (3 x 10^5^ cells per well). After 24 h of starvation, cells were treated with 5-FU for 4, 8, 12 h, and then incubated with **Y14** after adding 5-FU for 2 h each time. All cells were lysated and collected, and Coomassie Brilliant Blue method was used to quantify the proteins. The protein samples were separated by 10% SDS-PAGE and transferred to PVDF membranes (Millipore). Then the membranes were blocked for 1.5 h with TBST containing 5% (w/v) nonfat dry milk at room temperature. After washing with TBST, the membranes were incubated with the following antibodies: p-FGF Receptor 1 (Tyr766, Cell Signaling Technology), p-ERK (Cell Signaling Technology), p-AKT (Ser473, Cell Signaling Technology), β-Actin (Cell Signaling Technology), and GAPDH (Cell Signaling Technology). Subsequently, membranes were incubated with respective secondary antibody for 1 h. Protein bands were visualized using enhanced chemiluminescence kit (ECL; Bio-Rad). Data was processed using Image J software.

### MTT Assay

Human gastric cancer SGC-7901 and BGC-823 cells (4,000 cells/well) were seeded in a 96-well plate with RPMI-1640 medium for 24 h. Cells were treated with or without compounds (**Y14**, **Af23**, NDGA, 5-FU) under various concentrations for 72 h or the indicated time periods. And then 20 µl MTT solution (5 mg/ml) was added to the culture medium and incubated at 37°C for 4 h. At the end of incubation, produced crystal was dissolved in 150 µl dimethyl sulfoxide, and quantified at 490 nm using an enzyme-labeled meter (MD, USA). The IC_50_ values were calculated with GraphPad Prism 6.

### Colony Formation Assay

The gastric cancer cell lines (1,000 cells/well) were seeded in 6-well plates for 24 h, and then DMSO, **Y14**, **Af23**, NDGA and 5-FU was added to the culture medium at the indicated concentrations. After 8 or 24 h, the culture medium was replaced with RPMI-1640 medium that contained 10% FBS, and cells were cultured for another 9 days. Colonies were fixed with 4% paraformaldehyde for 15 min and stained with crystal violet staining solution. Visualized colonies were photographed and colonies containing more than 50 cells were counted using Image J software.

### Scratch-Wound Healing Assay

The 6-well culture dishes containing a monolayer of SGC-7901 and BGC-823 cells were starved of serum for 24 h, cells were then scratched using a sterile pipette tip forming a wound. Then cells were cultured in 2 ml growth medium in the presence of DMSO, **Y14** (2.5, 5, 10 µM), **Af23** (10 µM) or NDGA (10 µM). Images were taken using a microscopic camera system (Nikon, Tokyo, Japan) at indicated time points. The wound area was obtained by tracing the cell-free areas in the images using ImageJ software. The migration rate was expressed as the percentage reduction in wound area over time, which was calculated as follows (Martinotti and Ranzato, 2020):

$$Migration\;rate\;(\%)=\frac{Wound\;area_{0\;h}-Wound\;area_{T\;h}}{Wound\;area_{0\;h}}\times 100\%$$

where Wound area_0_ *_h_* is the wound area measured immediately after the scratch (time zero) and Wound area*_T_* *_h_* is the wound area measured at T hours after the scratch.

### Hoechst 33258 Staining

Human SGC-7901 and BGC-823 cells of logarithmic growth were seeded and incubated on sterile cover glasses for 24 h. All cells were treated with DMSO, **Y14** (5, 10, 20 µM), **Af23** (20 µM) or NDGA (20 µM) for 12 h. After fixed with 4% paraformaldelyde for 10 min at room temperature, cells were washed and stained with Hoechst 33258 (Beyotime) for 10 min. Then cells were observed under fluorescence microscopy (Nikon, Tokyo, Japan).

### Cell Cycle Analysis

Human SGC-7901 and BGC-823 cells (3 × 10^4^ cells/well) were seeded in a 6-well plate with RPMI-1,640 medium for 20 h. After 24 h of starvation, cells were treated with DMSO or 2.5 μM **Y14**. After 2 h of drug treatment, 40 ng/ml FGF2 was added to stimulate for 24 h. Cells were collected and fixed with 75% ice-cold ethanol for 24 h at -20 °C, then stained with 500 μl propidium iodide (PI) containing RNase A (BD Biosciences, San Diego, California, USA) for 15 min. The cell cycle distribution was determined using a FACS Calibur instrument (BD Biosciences Clontech) and the FlowJo program was used to analyze the data.

### Statistical Analysis

The results are presented in the mean ± SEM. The statistics were performed using a one-way ANOVA followed by the Dunnett test in Graph Pad Pro (Graph Pad, San Diego, CA, USA). A-value of *p* less than 0.05 (*p* < 0.05) was considered statistically significant.

## Results

### FGFR1 Over-Activation Correlated With Chemotherapy Resistance in Gastric Cancer Cell Lines

**Figure 1:**
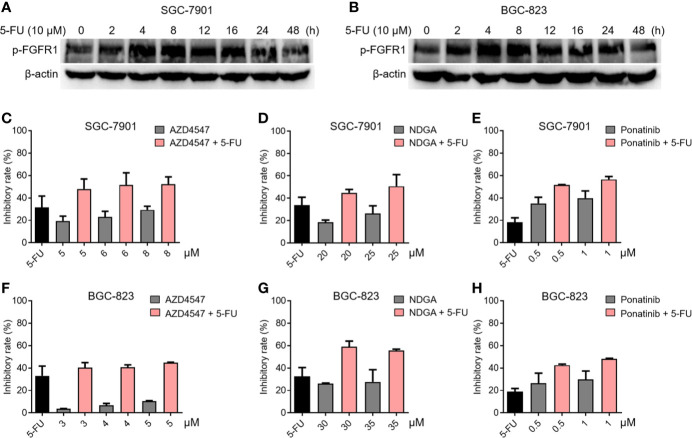
The relationship between FGFR1 and chemotherapy resistance in gastric cancer cells. The level of p-FGFR1 increased at early time points of 5-FU treatment in SGC-7901 **(A)** and BGC-823 cells **(B)**. The inhibitory rates of 5-FU (6.25 μM), FGFR1 inhibitors (AZD4547, NDGA or Ponatinib) or their combination treatment in SGC-7901 **(C–E)** and BGC-823 cells **(F–H)**.

To verify the relationship between FGFR1 and chemotherapy resistance in gastric cancer, the phosphorylation status of FGFR1 was analyzed in SGC-7901 and BGC-823 cells exposed to 5-fluorouracil (5-FU, 10 μM) for 48 h by Western blot. **Figures 1A, B** showed that the level of p-FGFR1 increased at early time points of 5-FU treatment, suggesting that gastric cancer cells exposed to 5-FU can lead to over-activation of FGFR1. Next, we explored whether 5-FU combined with FGFR1 inhibitors could increase the sensitivity of the SGC-7901 or BGC-823 cells to 5-FU. The inhibitory rates of 5-FU, FGFR1 inhibitors and their combination on gastric cancer cells were determined. As observed in **Figures 1C–H**, 5-FU combined with three FGFR1 inhibitors, AZD4547, NDGA, and ponatinib, significantly suppressed the growth ability of GC cell lines (including SGC-7901 and BGC-823). These results indicated that aberrant FGFR1 is highly relevant to 5-FU resistance and preventing the over-activation of FGFR1 can enhance the sensitivity of gastric cancer cells to 5-FU.

### Chemistry (Synthesis and Identification of Af23 Asymmetric Analogues)

**Figure 2:**
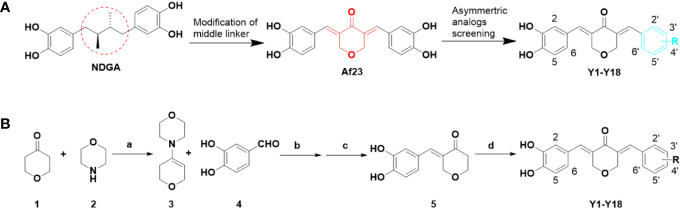
Design of novel asymmetric NDGA analogues as potential FGFR1 inhibitors **(A)** and the general synthesis routes of **Y1**–**Y18 (B)**. Reagents and conditions: (a) p-TSA, cyclohexane, reflux, 50%; (b) EtOH, 90°C, 30%; (c) 10% HCl solution, rt, 50%; (d) differently aldehydes, HCl gas, EtOH, 50–70°C, 10–70%.

**Figure 3:**
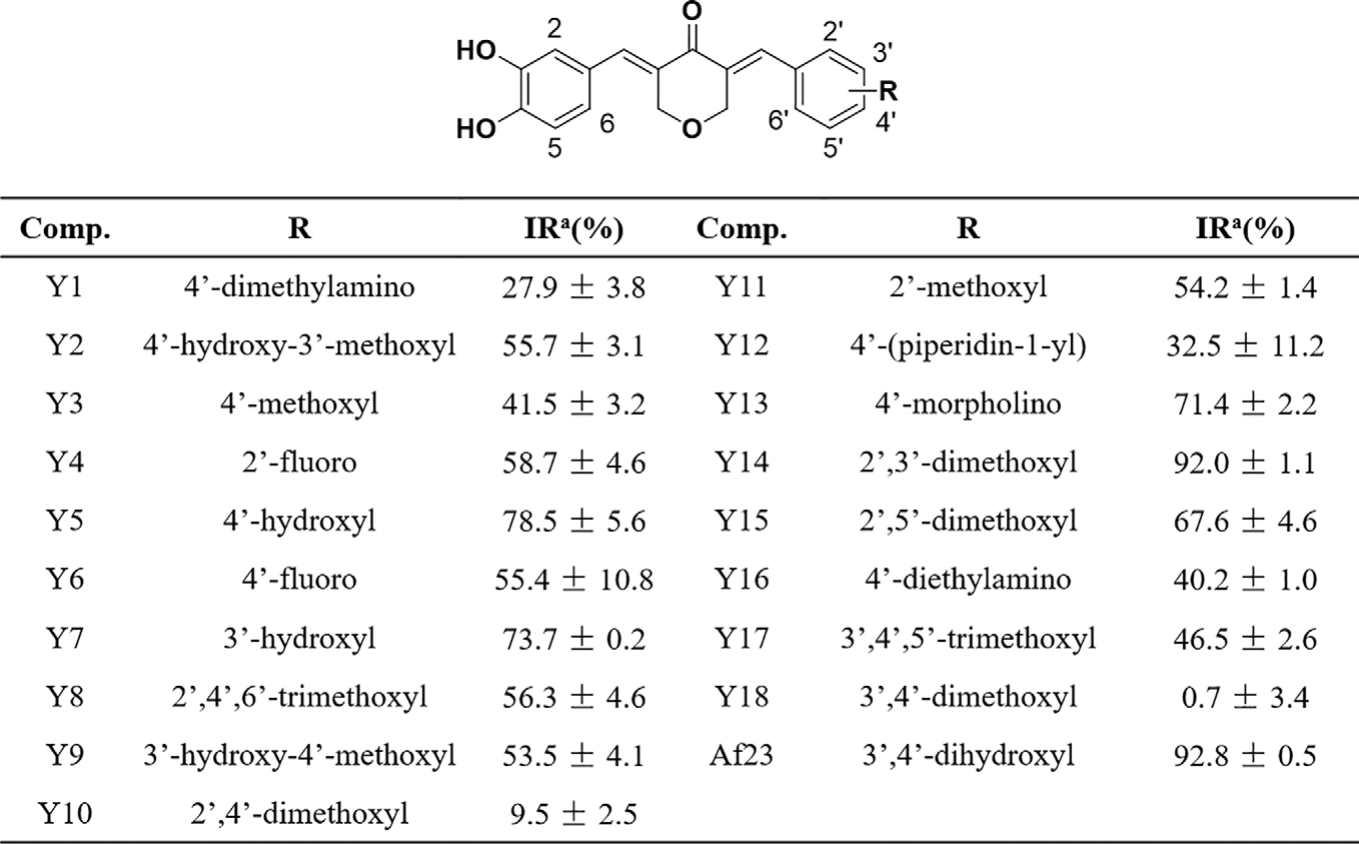
Chemical structures of the asymmetric NDGA derivatives and the corresponding inhibitory activities on FGFR1 kinase. IR^a^ means the inhibitory ratio of different compounds at the concentration of 20 μM.

The design and synthesis of the asymmetric derivatives **Y1–Y18** were shown in **Figures 2A, B**. The synthesis of **Y1–Y18** started from tetrahydro-4H-pyran-4-one (**1**), which was reacted with morpholine (**2**) to generate the enamines **3**. The compound **3** as an intermediate promoted the reaction between aldehyde and ketone in 1:1. Then, the ethanol solution of **3** with 3,4-dihydroxybenzaldehyde (**4**) was reacted under 78°C, adjusted the pH to acidic conditions to obtain the key intermediates **5**. Next, the solutions were condensed with different substituted benzaldehydes for preparing the desired **Y1–Y18**. All compounds were purified by column chromatography. The structures of synthetic compounds were shown in **Figure 3**, which were confirmed by electrospray ionization mass spectrometry (ESI-MS) and nuclear magnetic resonance spectroscopy (^1^H-NMR).

### The Inhibitory Activity of Synthetic Compounds on FGFR1 Kinase

Mobility shift assay was employed to measure the inhibitory activity of compounds on FGFR1 kinase. As shown in **Figure 3**, most of compounds can effectively inhibit FGFR1 kinase (inhibitory ratio, IR% > 50%) at a concentration of (20 µM). Among them, **Y14,** the most active compound, inhibited the activity of FGFR1 kinase by 92.0%, whose inhibitory effect was similar to that of lead compound **Af23** (IR%: **Af23**, 92.8%).

Some structure-activity relationship (SAR) information could be extracted based on compounds’ structure and their corresponding FGFR1 kinase inhibitory ratio. We observed that compounds **Y5** and **Y7** that retained a 4- or 3-hydroxyl on the right phenyl still displayed a favorable activity (IR%: **Y5**, 78.5%; **Y7**, 73.7%). Compounds **Y2** and **Y9**, the 4- or 3-methylated derivatives of **Af23**, also maintained at a moderate inhibition (IR%: **Y2**, 55.7%; **Y9**, 53.5%). We also found that, except for 4-morpholino group (IR%: **Y13**, 71.4%), introducing 4-nitrogen-containing substituent could strongly reduce the inhibitory activity (IR%: **Y1**, 27.9%; **Y12**, 32.5%; **Y16**, 40.2%). Noticeably, the FGFR1 inhibitory activity of compounds containing 4-methoxyl group decreased to some extent. (IR%: **Y3**, 41.5%; **Y10**, 9.5%; **Y18**, 0.7%), whereas those derivatives containing 2-methoxyl group exhibited good inhibition effect (IR%: **Y11**, 54.2%; **Y14**, 92.0%; **Y15**, 67.6%). Overall, different substituents on the right benzene ring have different effects on inhibitory activity FGFR1 kinase, and this SAR information can provide valuable information for further modification.

### Molecular Docking and Molecular Dynamics Simulation

**Figure 4:**
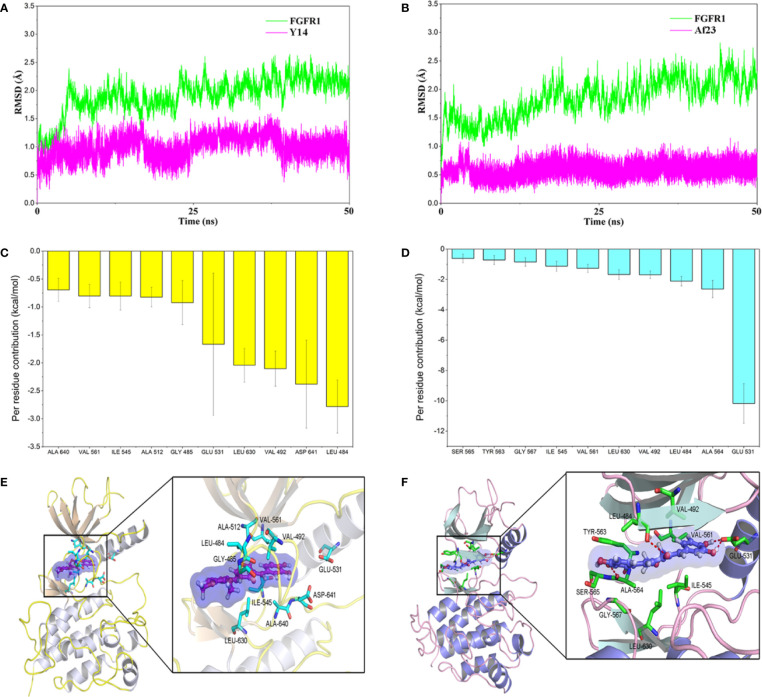
Molecular docking and molecular dynamics simulation of **Y14** or **Af23** to the activity cavity of FGFR1. **(A, B)** Time evolution of the root-mean-square deviation (RMSD) of FGFR1/**Y14** complex and FGFR1/**Af23** complex. **(C, D)** Per-residue of top 10 contribution to the binding free energy of FGFR1/**Y14** complex (**C**, Yellow) and FGFR1/**Af23** complex (**D**, light blue). **(E, F)** Molecular docking of **Y14** **(E)** and **Af23** **(F)** with FGFR1 kinase. Red dashed lines represented intermolecular hydrogen bonds.

Molecular docking was firstly used to investigate the possible binding modes of **Y14** or **Af23** with FGFR1. According to our previous established method (Ying et al., 2017), each compound was docking with the crystal structure of FGFR1, including 3RHX, 4UWC, and 5EW8. The most probable binding conformation which displayed the lowest binding energy between inhibitor and protein (**Table 1**, 4UWC, -9.49 kcal/mol for **Y14** and -9.1 kcal/mol for **Af23**) was selected as input structure for the subsequent molecular dynamics (MD) simulations. The results (**Figures 4A**, **B**) showed that the RMSD of the receptor in FGFR1/**Y14** and FGFR1/**Af23** complexes were converged after 5–10 and 15–20 ns in 50 ns MD simulations respectively, and the RMSD of backbone atoms both were 1.5–2.5 Å. Besides, binding free energies were calculated using MM/GBSA method by picking 500 snapshots from the last 10 ns stable trajectories and then decomposed into inhibitor-residue interaction. As **Figure 4C** shown, the top 10 residues contributing to the binding free energy of **Y14**/FGFR1 were ranked in the following order: LEU484, ASP641, VAL492, LEU630, GLU531, GLY485, ALA512, ILE545, VAL561, ALA640 (**Figure 4C**). Among the top 10 residues, 80% of them belonged to hydrophobic amino acid residue, which indicated that the interaction between **Y14** and FGFR1 was likely to be hydrophobic interaction. While **Figure 4D** showed that GLU531 contributed the dominated interaction to the FGFR1/**Af23** complex. To characterize and estimate the details of action mode of **Y14** and **Af23**, last snapshot of FGFR1/**Y14** and FGFR1/**Af23** in 50 ns MD simulations was analyzed with PyMOL software. **Figures 4E, F** intuitively presented the differences in action patterns of **Y14** and **Af23**, respectively. **Y14** was surrounded by hydrophobic clusters formed by the first 10 residues mentioned above and did not form hydrogen bonds with the surrounding residues. On the contrary, **Af23** mainly formed four hydrogen bonds with GLU531, ALA564 and LEU484 (**Figure 4F**), which were the top three residues contributing to the binding free energy of **Af23**/FGFR1 (**Figure 4D**). Taken together, the potential interaction mechanisms of **Y14/**FGFR1 and **Af23/**FGFR1 complexes were discussed by combining molecular docking and molecular dynamics simulation, which provided considerable information for the design of novel FGFR1 inhibitors.

**Table 1:** The docking score of different FGFR1 crystal structures with **Y14** and **Af23**.

FGFR1 crystal structure(PDB ID)	Docking score (kcal/mol)	
	Y14	Af23
3RHX	-8.33	-8.30
4UWC	-9.49	-9.10
5EW8	-8.52	-8.65

### Y14 Inhibited FGF2-Induced Phosphorylation of FGFR1 and Its Downstream Signaling in Gastric Cancer Cells

**Figure 5:**
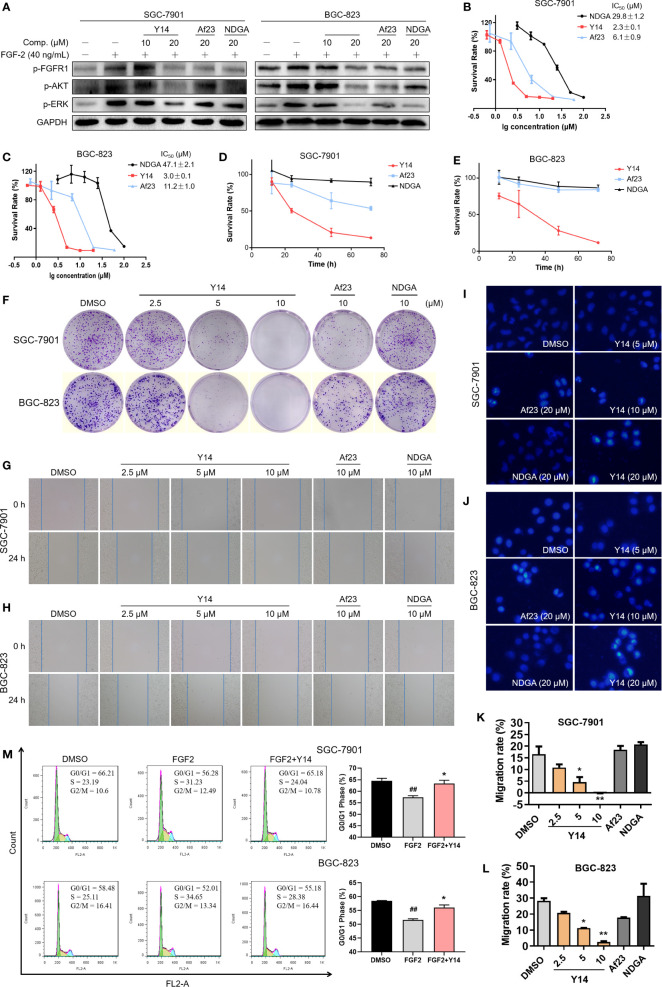
**Y14** inhibited the cellular FGFR1 phosphorylation and exhibited anti-cancer effect on gastric cancer cells. **(A)** The p-FGFR1, p-AKT, and p-ERK levels were analyzed by Western blot. SGC-7901 and BGC-823 cell lines were incubated with compounds for 2 h before stimulation with FGF-2 for 15 min. **(B, C)** The IC_50_ values were determined on SGC-7901 and BGC-823 cell lines with different concentrations of NDGA (100, 50, 25, 12.5, 6.25, 3.125 μM) or **Y14** (20, 10, 5, 2.5, 1.25, 0.625 μM) or **Af23** (60, 20, 6.667, 2.222, 0.741 μM) for 72 h. **(D, E)** The cells were incubated with compounds (5 μM) for 12, 24, 48, and 72 h, and the survival rate was determined by MTT assay. **(F)** **Y14** suppressed the proliferation of SGC-7901 and BGC-823 cell lines. Cells were incubated with compounds for 8 h. Then the medium was changed, and cells were cultured for about 9 days. (**G, H** and **K, L**) **Y14** suppressed the migration of gastric cancer cells. Scratch wound-healing assays were performed using SGC-7901 and BGC-823 cells, and these cells were treated with compounds for 24 h. *p < 0.05, **p < 0.01 *vs.* DMSO. The apoptotic cells induced by **Y14** in SGC-7901 **(I)** or BGC-823 **(J)** cells were determined by Hoechst 33258 staining assay. **(M)** Cell cycle distribution was measured by flow cytometry. ^##^ *p* < 0.01 *vs.* DMSO; *p < 0.05 *vs.* FGF2.

To confirm the inhibition of **Y14** on cellular FGFR1 phosphorylation, the inhibitory effect of **Y14** on FGF2-induced FGFR1 phosphorylation was detected by Western blot analysis in gastric cancer cells. **Figure 5A** showed that **Y14** displayed significant inhibitory effect on FGF-2 induced phosphorylation of FGFR1 in SGC-7901 and BGC-823 cells with a dose-dependent manner. Furthermore, we observed that pretreatment with **Y14** for 2 h significantly suppressed FGF2- induced activation of the main downstream effectors of FGFR1, such as p-ERK and p-AKT (**Figure 5A**). It is suggested that **Y14** may have anti-cancer and chemosensitizing effects on gastric cancer by inhibiting FGFR1 phosphorylation and its downstream signaling pathway.

### Y14 Suppressed Cell Migration, Inhibited Cell Growth Through Cell Cycle Arrest and Apoptosis

To assess the effect of **Y14** on the growth of gastric cancer cells, both SGC-7901 and BGC-823 cells were exposed to **Y14** at different concentrations or at different times. By comparison with lead compounds, **Af23** and NDGA, **Y14** showed an evident inhibitory effect on the growth of gastric cancer cells with IC_50_ values of 2.3 μM for SGC-7901 and 3.0 μM for BGC-823 cells. (**Figures 5B, C**). **Y14** had a time-dependent inhibitory effect on GC cells within 72 h, and the inhibitory effect was better than that of the leading compounds **Af23** and NDGA at the same concentration (**Figures 5D, E**). Moreover, colony formation assay indicated that **Y14** significantly reduced the colony formation of SGC-7901 and BGC-823 cells in a concentration-dependent manner (**Figure 5F**). Tumor metastasis is the most important prognostic factor for survival of patients. Our results showed that **Y14** inhibited the migration of SGC-7901 cells in a dose-dependent manner, while the lead compounds **Af23** and NDGA had almost no inhibitory effect on the migration of SGC-7901 cells (**Figures 5G, K**). The similar results were observed in BGC-823 cells (**Figures 5H, L**). In addition, apoptosis and cell cycle analysis were carried out to explore how **Y14** affects the growth and survival of gastric cancer cells. On one hand, the morphological changes of nucleus after Hoechst 33258 staining were observed by fluorescence microscope to determine the effect of **Y14** on apoptosis of gastric cancer cells. As shown in **Figures 5I, J**, obvious chromatin condensation (strong blue fluorescence) and nuclear fragmentation were observed after 12 h treatment with **Y14** at concentrations of 10 and 20 μM. On the other hand, the effect of **Y14** on cell cycle of gastric cancer was detected by flow cytometry. The results are displayed in **Figure 5M** and showed that 2.5 μM **Y14** induced the accumulation of G0/G1 phase in both SGC-7901 and BGC-823 cells. Taken together, these results indicated that **Y14** not only suppressed the migration of gastric cancer cells, but also suppressed the growth and survival of gastric cancer cells by inducing apoptosis and inhibiting cell cycle progression from G0/G1 to S phase.

### Y14 Enhanced Chemosensitivity of Gastric Cancer Cells

**Figure 6:**
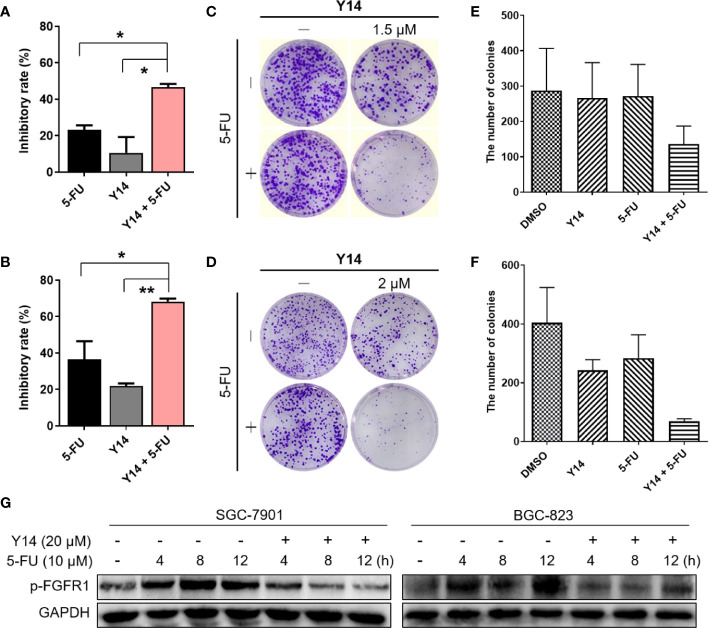
**Y14** enhanced 5-FU-induced cytotoxicity in SGC-7901 and BGC-823 cells. **(A)** The inhibitory rate of SGC-7901 cells after incubating 5-FU (10 μM), **Y14** (1.5 μM) or their combination for 72 h. *p < 0.05. **(B)** The inhibitory rate of BGC-823 cells after incubating 5-FU (6.25 μM), **Y14** (2 μM) or their combination for 72 h. *p < 0.05 and **p < 0.01. **(C)** The effects of 5-FU (6.25 μM), **Y14** (1.5 μM) or their combination on the ability of SGC-7901 cells to form colonies. Cells were incubated with compounds for 24 h. The number of colonies in each well were counted and displayed in **(E)**. **(D)** The effects of 5-FU (6.25 μM), **Y14** (2 μM) or their combination on the ability of BGC-823 cells to form colonies. Cells were incubated with compounds for 24 h. The number of colonies in each well were counted and displayed in **(F)**. **(G)** The p-FGFR1 levels were analyzed by Western blot. After 24 h of starvation, SGC-7901 and BGC-823 cells were treated with 10 μM 5-FU for 4, 8, 12 h, and then incubated with 20 μM **Y14** after adding 5-FU for 2 h each time.

To evaluate whether **Y14** could enhance chemosensitivity of gastric cancer cells, we tested the effect of **Y14** in combination with 5-FU, the first-line chemotherapy for gastric cancer. As shown in **Figures 6A–F**, the combination of the two drugs can dramatically inhibit the growth of gastric cancer cells, indicating the addition of **Y14** enhanced the cytotoxic effect of the 5-FU against SGC-7901 and BGC-823 cells. MTT assay showed that the inhibition rate of 5-FU combined with **Y14** raised significantly (**Figures 6A, B**), and synergistic effects were observed on SGC-7901 (q = 1.49) and BGC-823 (q = 1.35) cells. Colony formation assay showed that 5-FU combined with **Y14** formed fewer colonies (**Figures 6C–F**), which had synergistic effects on SGC-7901 (q = 4.23) and BGC-823 (q = 1.43) cells. Furthermore, to investigate whether **Y14** enhances the chemosensitivity of 5-FU to gastric cancer cells by inhibiting FGFR1, we detected the activation status of FGFR1 in gastric cancer cells after the combination of **Y14** and 5-FU. As shown in **Figure 6G**, compared with the cells treated with 5-FU alone (4, 8, and 12 h), combined treatment with 5-FU and **Y14** significantly down-regulated the level of FGFR1 phosphorylation in gastric cancer cells (**Figure 6G**). Collectively, these results suggested that **Y14**, as an effective inhibitor of FGFR1, synergistically enhanced the anti-cancer effect of 5-FU by inhibiting FGFR1 phosphorylation in two gastric cancer cell lines.

## Discussion

In recent years, the relationship between FGFR1 and gastric cancer has become increasingly apparent. Several reports have shown that FGFR1 mRNA and protein were highly expressed in cancer tissues of patients with gastric cancer and could result in distant metastasis and recurrent disease (Shin et al., 2000; Inokuchi et al., 2017). It is also found that FGFR1 was significantly up-regulated in drug-resistant gastric cancer cell lines (Chen et al., 2014). Anyhow, the high expression of FGFR1 in gastric cancer was tightly associated with the poor survival rate of patients with gastric cancer (Schafer et al., 2015; Inokuchi et al., 2017). Recently, selective inhibitors of the FGFRs have been developed and some of them are under phase study in patients with gastric cancer or advanced Gastrointestinal Stromal Tumor (GIST), such as AZD4547 (NCT01795768), JNJ-42756493 (NCT02699606) and BGJ398 (NCT02257541). Besides, our group also discovered some favorable FGFR1 inhibitors **L16H50** (Wu et al., 2017a), **L6123** (Xu et al., 2015), and **Af23** (Wu et al., 2015), which exhibited effective anti-gastric cancer effects *in vitro*. These results suggested that FGFR1 may be a new target for the treatment of gastric cancer and FGFR1 inhibitors could be used as monotherapy or in combination with chemotherapy.

Natural products, the source of almost all medicinal preparations in ancient times, are still a rich source of models and templates for drug discovery and development. Detailed analysis of all small-molecule drugs approved by the Food and Drug Administration (FDA) from 1981 to 2014 revealed that 65% of those drugs were either natural products or natural product derivatives or natural product-based synthetic mimics (Newman and Cragg, 2016). Furthermore, over 80% of all small-molecule anti-cancer drugs are classified as natural product-based agent, suggesting that novel compounds from natural sources would be considered as a safe and effective strategy for the discovery of anti-cancer drugs. Nordihydroguaiaretic acid (NDGA), a natural lignan compound, has been reported to have potential medical applications in multiple diseases, including metabolic disorders (Chan et al., 2018), virus infection (Soto-Acosta et al., 2014; Merino-Ramos et al., 2017), cancers (Huang et al., 2015; Mundhe et al., 2015), and neurological disorders (Liu et al., 2012). Meso-NDGA (masoprocol, Actinex) was previously approved as a local antineoplastic drug for actinic keratosis in the United States (Olsen et al., 1991), but was subsequently withdrawn due to its low market demand and contact sensitivity. Terameprocol, also known as tetra-O-methyl nordihydroguaiaretic acid, M4N and EM-1421, is a derivative of NDGA and is currently undergoing Phase I trials for treating patients with recurrent high grade glioma (NCT02575794) (Grossman et al., 2012; Tibes et al., 2015). It is reported that the underlying anti-cancer mechanisms of NDGA was to inhibit a series of cancer related signaling molecules, such as IGF-1R, HER2, and mTORC1 (Zavodovskaya et al., 2008; Zhang et al., 2012). In the previous study, we found that the inhibitory activity of NDGA on FGFR1 kinase was superior to that of FGFR3. Subsequently, it was found that a symmetrically structured NDGA derivative of **Af23** could effectively inhibit FGFR1 kinase in a non-ATP competitive manner (IC_50_ = 1.4 μM) and had potent anti-cancer activity against human lung cancer *in vitro* and *in vivo*. In this study, we designed a series of **Af23** asymmetric analogues by retaining the basic structural framework (bisaryl-1,4-dien-3-one), as well as the unilateral active functional groups (3,4-dihydroxyl), and obtain the compounds **Y1–Y18**. Due to the existence of two CH=C in compounds **Y1–Y18**, the isomeric type of these compound was uncertain. We analyzed the chemical shifts of two β hydrogens (Ar-CH=C, Ar’-CH=C) in compounds. Firstly, it was found that the precursor compound **5** was E-stereoisomers because the ^1^H-NMR spectrum showed the chemical shift of β hydrogen (Ar-CH=C) in compound **5** was 7.435 PM. Then, we found that the hydrogen chemical shift of Ar-CH=C in compounds **Y1**–**Y18** was about 7.5 PM, indicating that the side of these compounds was E-stereoisomers. Subsequently, the chemical shift of Ar’-CH=C was also found to be around 7.5 PM and relatively close Ar-CH=C. Therefore, compounds **Y1**–**Y18** were considered to be EE-stereoisomers.

Next, Mobility shift assay was employed to screen and **Y14** was found to have the highest inhibitory activity against FGFR1. The inhibitory activity of **Y14** on FGFR1 kinase was similar to that of **Af23** (92.0 *vs* 92.8%), but the inhibitory effect of **Y14** on gastric cancer cells was stronger than that of **Af23**. On the one hand, this may be due to the increased lipophilicity of **Y14**, which promotes its ability to penetrate cell membranes. Furthermore, to understand the interaction between **Y14** and FGFR1, the results of molecular docking and molecular dynamics simulation suggested that **Y14** could bind to FGFR1 kinase through hydrophobic interaction rather than hydrogen bond or covalent bond interactions, which is quite different from the interaction mode of **Af23** and FGFR1. The interaction between **Y14** and FGFR1 is highly similar to that between ARQ069 and FGFR1, which was characterized as an ATP-independent FGFR1 inhibitor that targets the auto-inhibited, inactive conformation of FGFR1 (Eathiraj et al., 2011). It is noteworthy that small changes in substituents on the benzene ring of **Af23** resulted in a completely different mode of interaction with FGFR1, suggesting that asymmetric analogues design strategy might be a feasible means to discover new types of TKIs.

It is reported that both mRNA and protein expression of FGFR1 are high in gastric cancer tissues. We also found that the expression of FGFR1 is significantly increased after 5-FU resistance in gastric cancer cells. Hence, next efforts were made to clarify whether **Y14** can exert anti-gastric cancer effect and sensitize gastric cancer cells to chemotherapeutic drugs by inhibiting FGFR1. Western blot analysis of the phosphorylated FGFR1 showed that **Y14** inhibited the phosphorylation of FGFR1 in gastric cancer cells in a dose-dependent manner. And FGF2-induced activation of the main downstream effectors of FGFR1, such as p-ERK and p-AKT, was also suppressed after pretreatment with **Y14** for 2 h. Next, MTT assay and colony formation assay showed that **Y14** significantly inhibited the growth of gastric cancer cells, and **Y14** was observed to induce cell apoptosis and cell cycle arrest in gastric cancer cell lines. Besides, **Y14** significantly inhibited the migration of gastric cancer cells. More importantly, the anti-gastric cancer activity of **Y14** was dose-dependent, and its effect was significantly stronger than that of **Af23** and NDGA. Encouraged by its promising FGFR1 inhibitory potency, we attempted to evaluate whether **Y14** combined with 5-FU could enhance the sensitivity of gastric cancer cells to 5-FU. The results showed that **Y14** synergistically enhanced the anti-cancer effect of 5-FU by inhibiting the viability and colony formation of gastric cancer cells. Further, WB analysis revealed that the combination of 5-FU and **Y14** significantly reduced FGFR1 phosphorylation in gastric cancer cells compared with those treated with 5-FU alone. Taken together, these results confirm that **Y14** exerts anti-gastric cancer activity and enhances the sensitivity of gastric cancer cell to 5-FU by inhibiting the phosphorylation of FGFR1.

In conclusion, **Y14**, a promising inhibitor of FGFR1, was obtained by using **Af23** and NDGA as lead compounds. By inhibiting FGFR1 phosphorylation and its downstream signaling pathway in gastric cancer cells, it inhibited the growth, migration, and survival of cancer cells, as well as enhancing the chemosensitivity of cancer cells to 5-FU. This study provides a potential FGFR1 inhibitor for the treatment of gastric cancer and provides a reference for the discovery of novel FGFR1 inhibitors from the sources of bioactive natural products.

## Data Availability Statement

The raw data supporting the conclusions of this article will be made available by the authors, without undue reservation, to any qualified researcher.

## Author Contributions

WL, JW, and QC contributed to the conception of the study. JW, JX, QC, MZ, and DY contributed to the data acquisition and analysis. JX and JW performed the synthesis of compounds. MZ, DY, QC, ZD and FK performed the cell experiment. WF and QC performed molecular docking and molecular dynamics. QC, JX, MZ, and WF were responsible for drafting the manuscript, and all authors were involved in reviewing and revising it critically. WL, LW, TW, ZL, and PQ provided important intellectual and technical contribution.

## Funding

This work was supported by the National Natural Science Foundation of China (Grant No. 81803580, 81803421, 81773579); the Zhejiang Province Natural Science Fund of China (Grant Nos. LY19H310003, LGF20H310003, LQ18H280008); and Natural Science Foundation of Wenzhou City (Grant No. Y20170158). The Opening Project of Zhejiang Provincial Top Key Discipline of Pharmaceutical Sciences.

## Conflict of Interest

The authors declare that the research was conducted in the absence of any commercial or financial relationships that could be construed as a potential conflict of interest.
